# Supplementary material for: Transcription Factor and miRNA Interplays Can Manifest the Survival of ccRCC Patients
Source: Cancers (Basel). 2019 Oct 28;11(11):1668. doi: 10.3390/cancers11111668 (PMC6895828; doi:10.3390/cancers11111668)
Supplement: Supplementary file 1 [file cancers-11-01668-s001.zip › cancers-616468-suppl proof.docx]

Supplementarial Materials: Transcription Factor and miRNA Interplays Can Manifest the Survival of ccRCC Patients


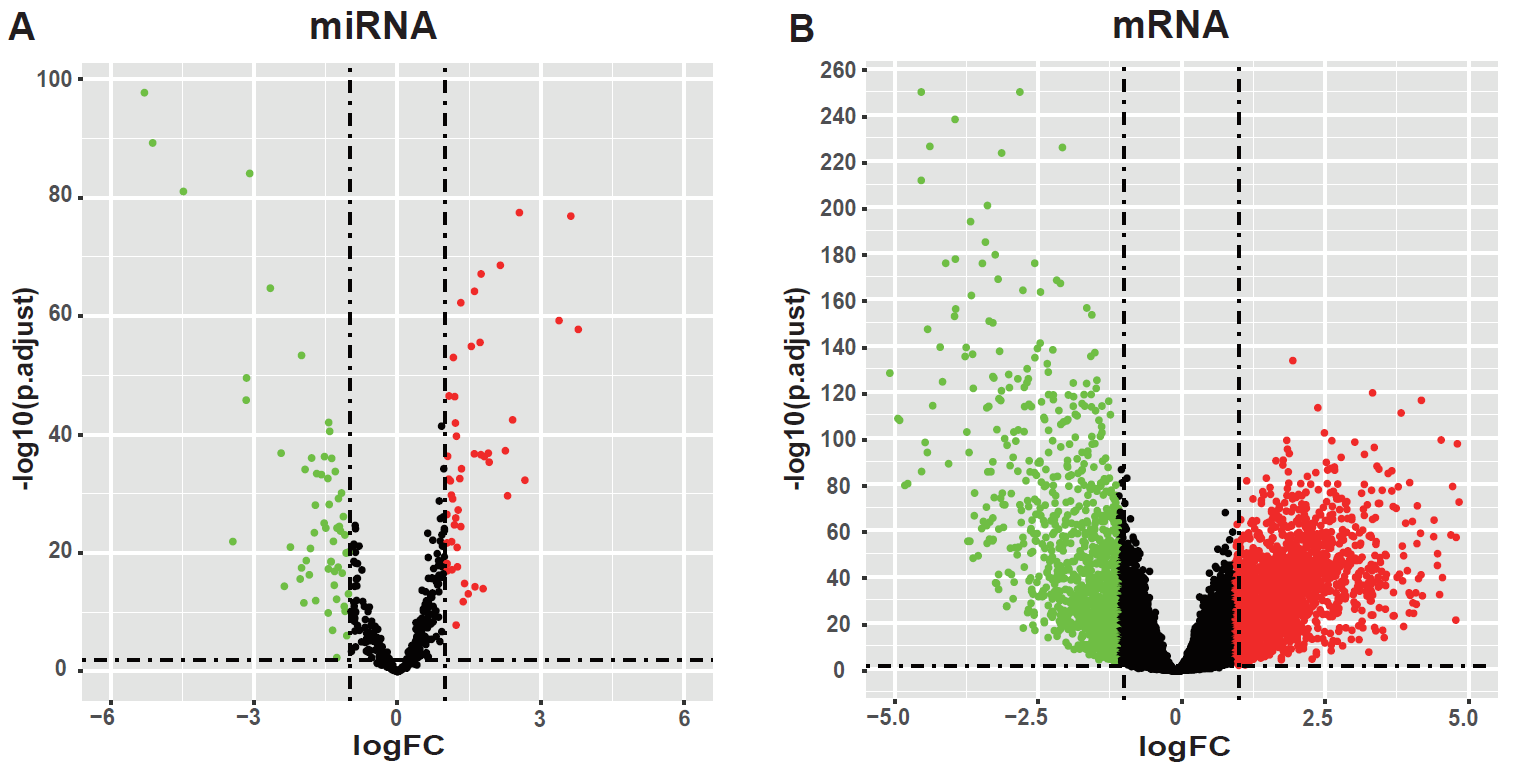


**Figure S1.** The volcano map of differentially expressed genes and miRNAs. The volcano map of differentially expressed miRNA (**A**) and mRNA (**B**). The red point represents up-regulated expression and the green point represents down-regulated expression with statistical significance (*p*-value < 0.05).


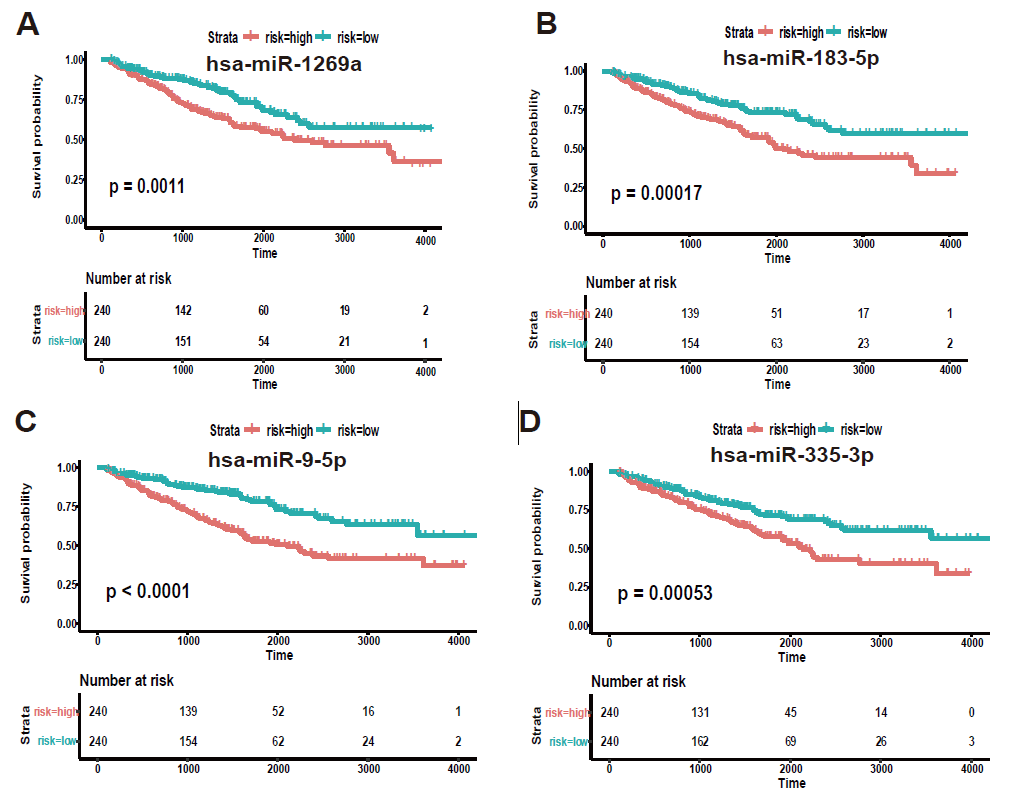


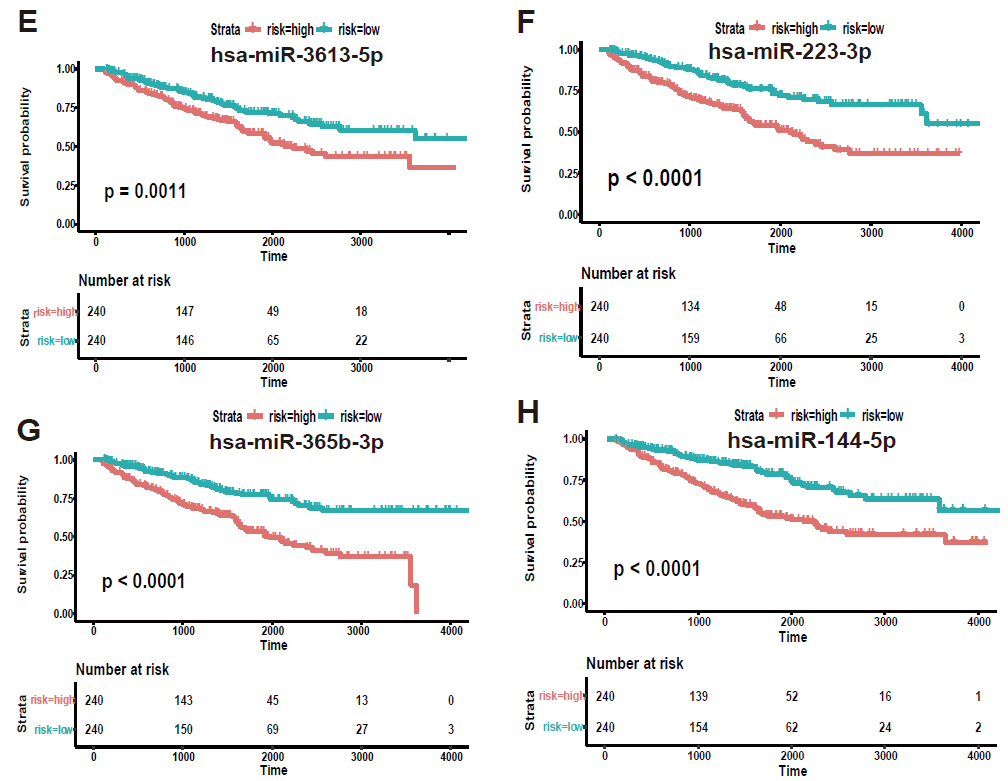


**Figure S2.** Kaplan-Meier survival based on the risk score of eight miRNAs. Overall survival curves of high-risk and low-risk groups for hsa-miR-1269a (**A**), hsa-miR-183-5p (**B**), hsa-miR-9-5p (**C**), hsa-miR-335-3p (**D**), hsa-miR-3613-5p (**E**), hsa-miR-223-3p (**F**), hsa-miR-365b-3p (**G**), hsa-miR-144-5p (**H**).


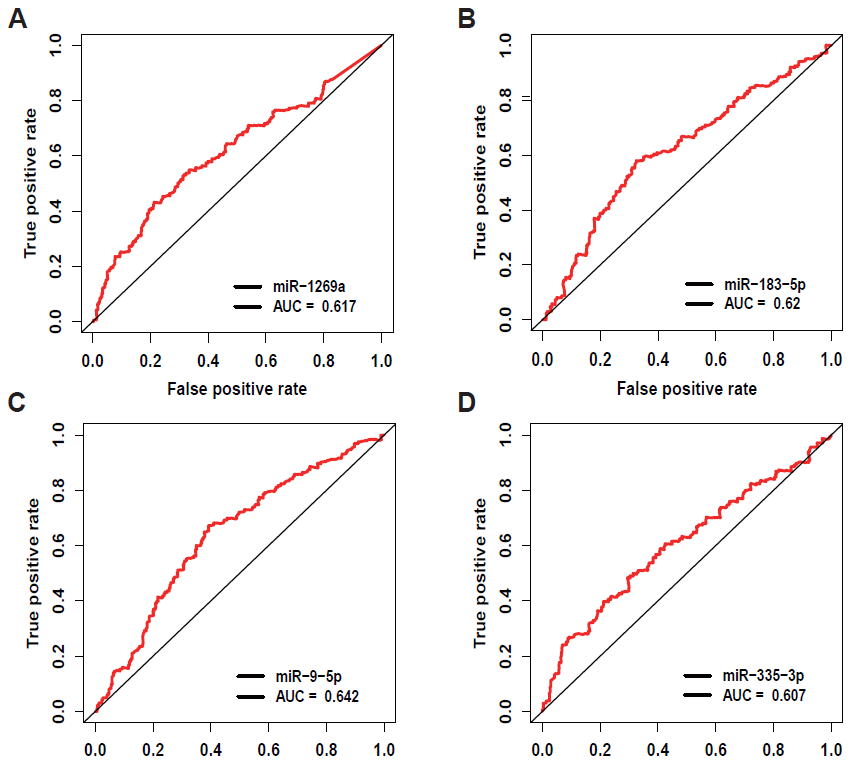


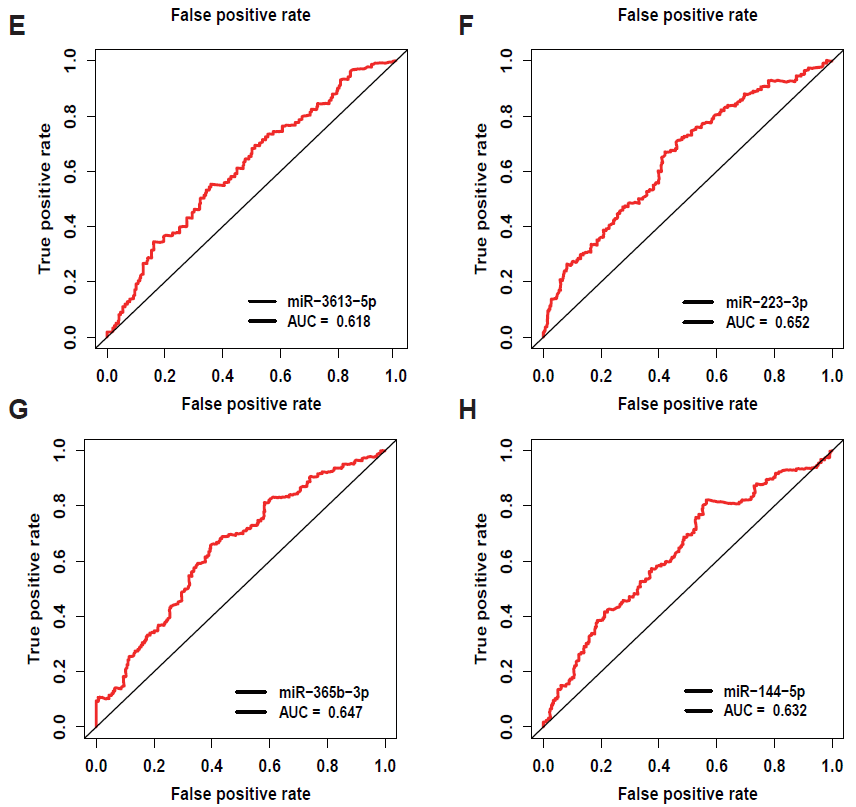


**Figure S3.** ROC curves based on the risk score of eight miRNAs. ROC curves for high and low risk of hsa-miR-1269a (**A**), hsa-miR-183-5p (**B**), hsa-miR-9-5p (**C**), hsa-miR-335-3p (**D**), hsa-miR-3613-5p (**E**), hsa-miR-223-3p (**F**), hsa-miR-365b-3p (**G**), hsa-miR-144-5p (**H**).


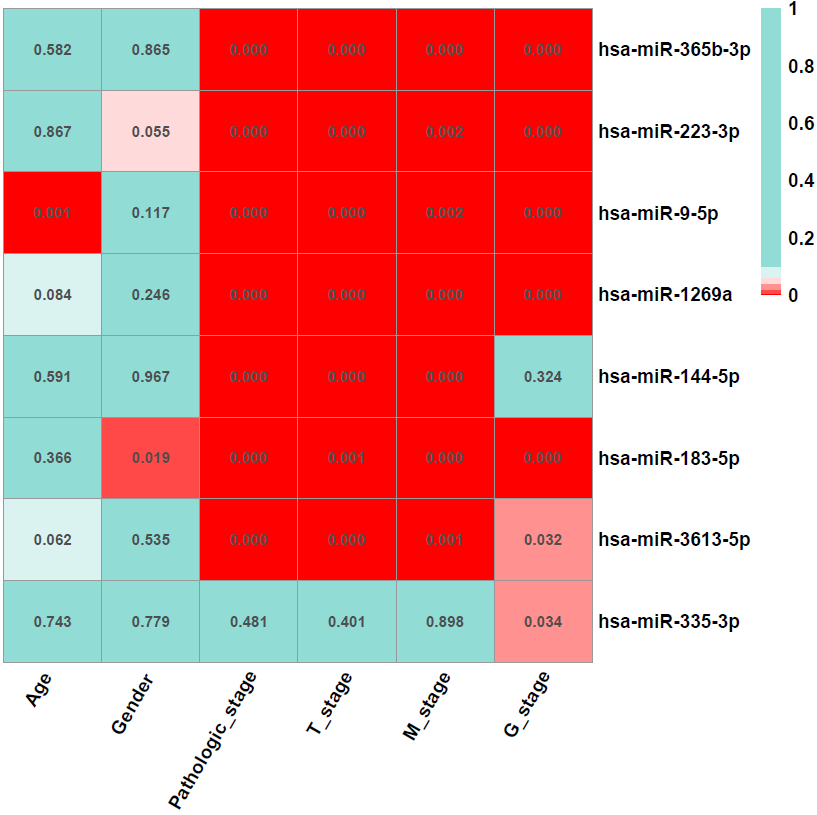


**Figure S4.** The correlation between the expression level of eight prognostic miRNAs and clinical factors. The correlation heat map between clinical parameters and prognostic miRNA expression levels in ccRCC. The red box represents a *p*-value < 0.05; the green box represents a *p*-value > 0.05.


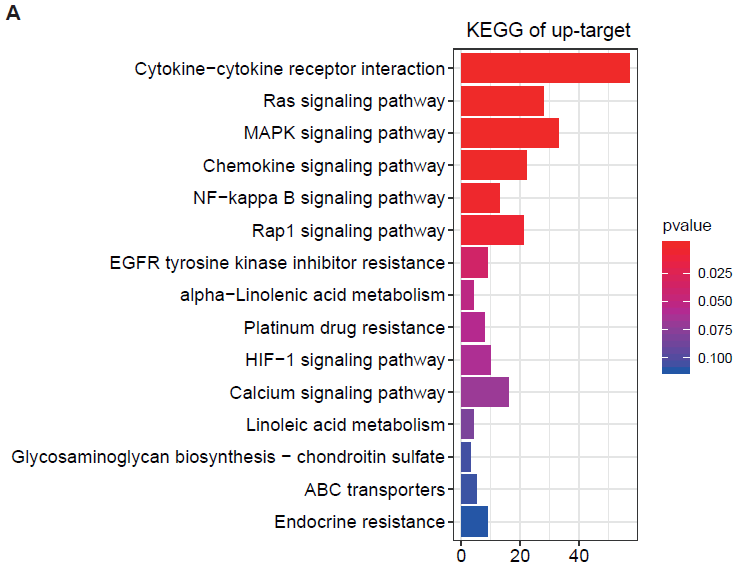


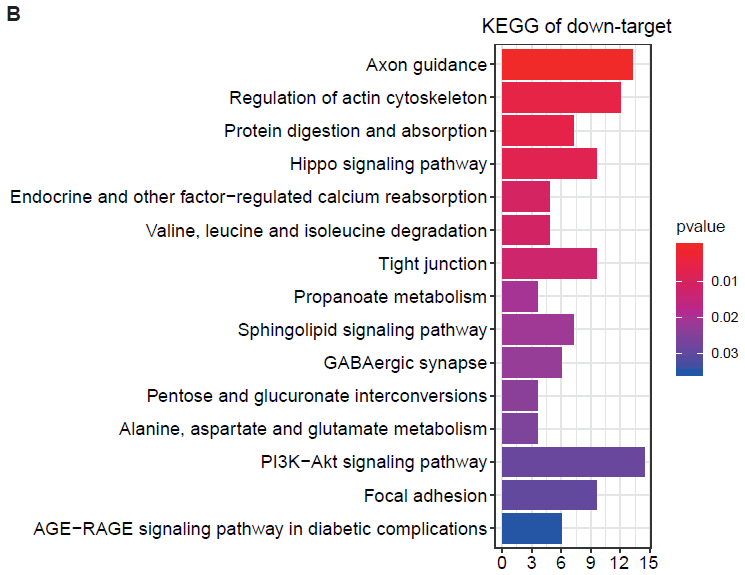


**Figure S5.** The KEGG signaling pathway analysis of up-regulated and down-regulated targets. Signaling pathways of KEGG enrichment of up-regulated targets(**A**) and down-regulated targets(**B**).


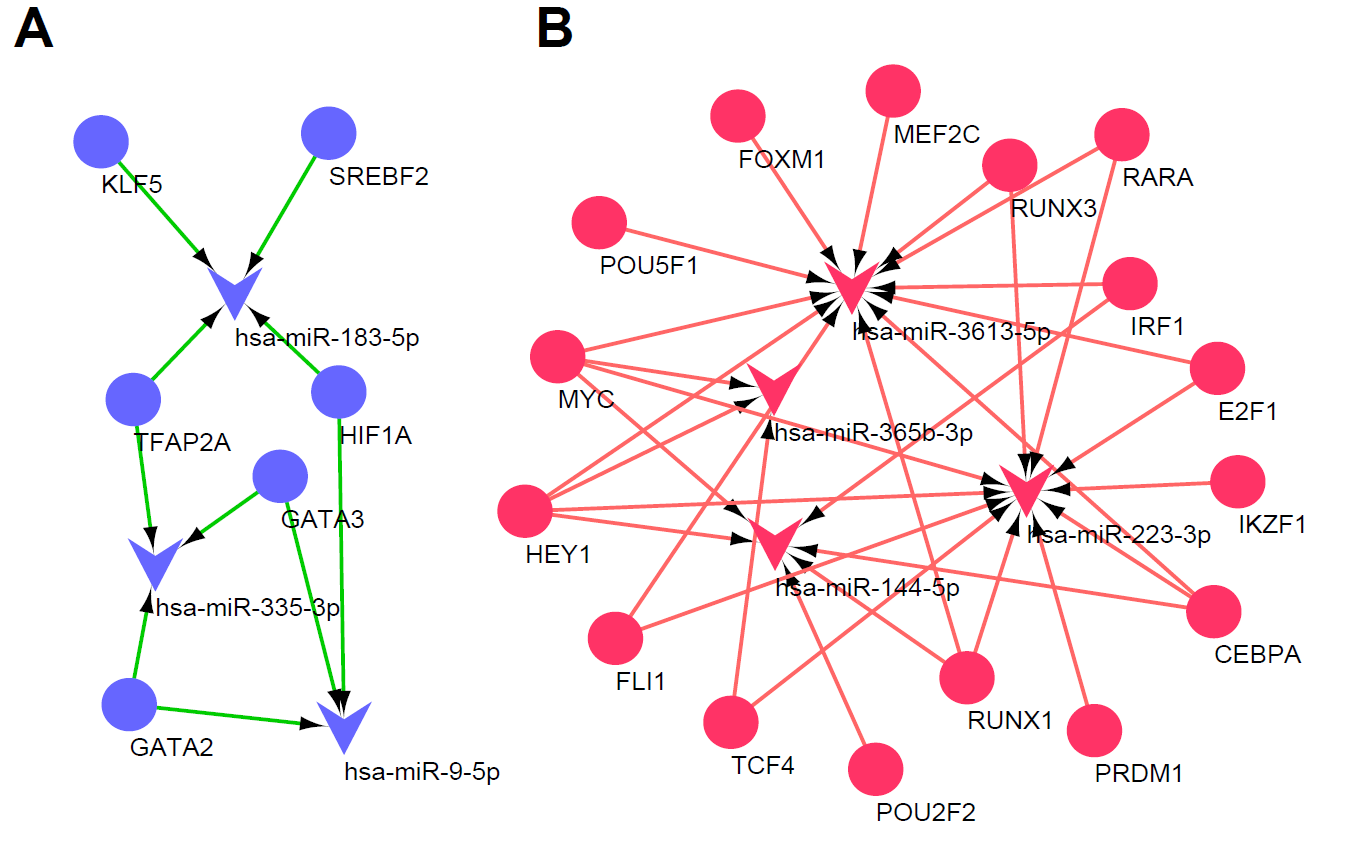


**Figure S6.** Transcription factors regulating eight miRNAs. Six down-regulated transcription factors down-regulate three down-regulated miRNAs (**A**); sixteen up-regulate transcription factors up-regulate four up-regulated miRNAs (**B**). The red represents up-regulation of gene expression and the blue represents down-regulation of gene expression.
