# Supplementary figures and images for: Transcription Factor and miRNA Interplays Can Manifest the Survival of ccRCC Patients
Source: Cancers (Basel). 2019 Oct 28;11(11):1668. doi: 10.3390/cancers11111668 (PMC6895828; doi:10.3390/cancers11111668)

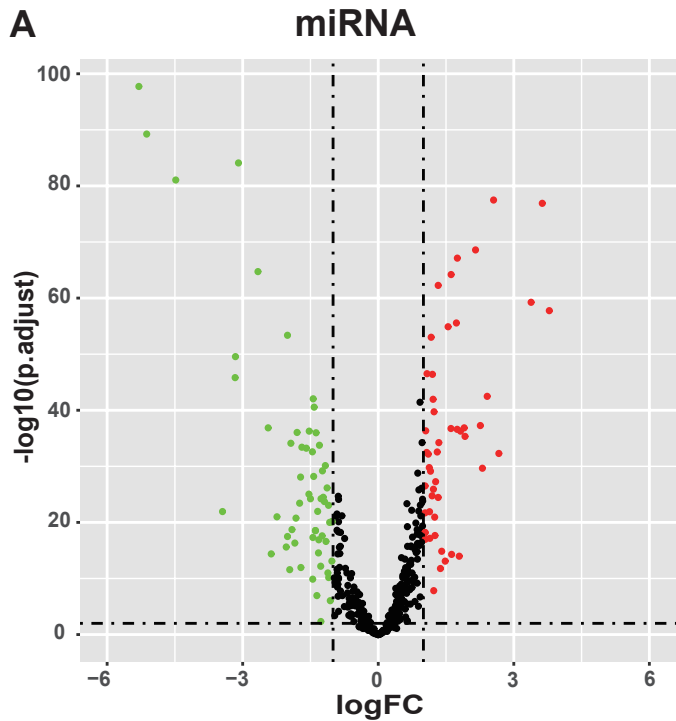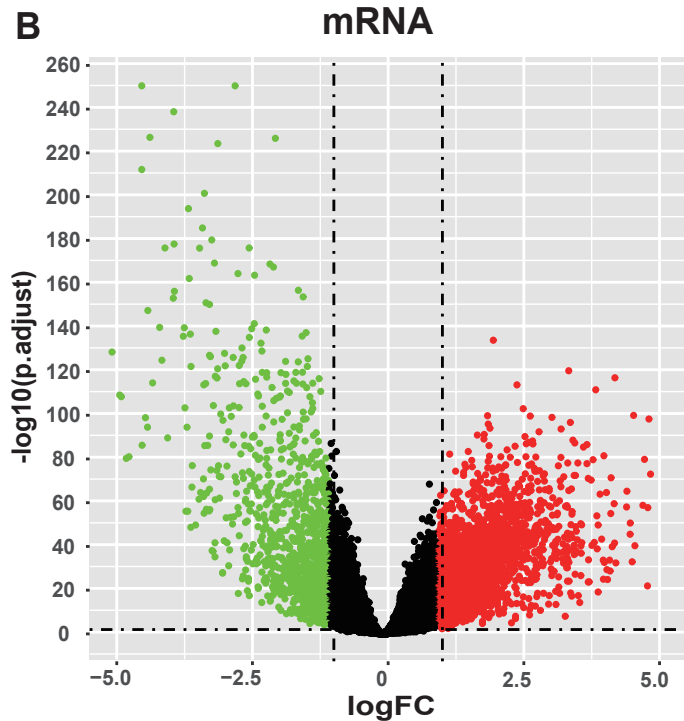

Supplement: Supplementary file 1 [file cancers-11-01668-s001.zip › Figure S1.pdf]

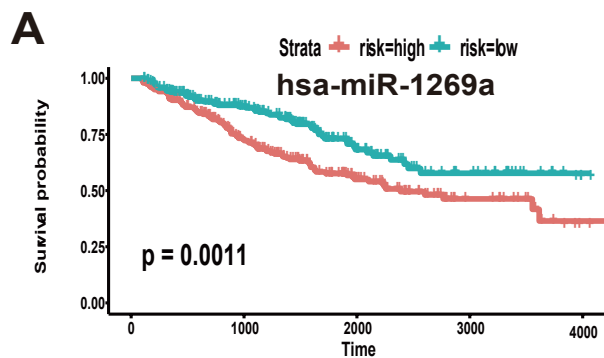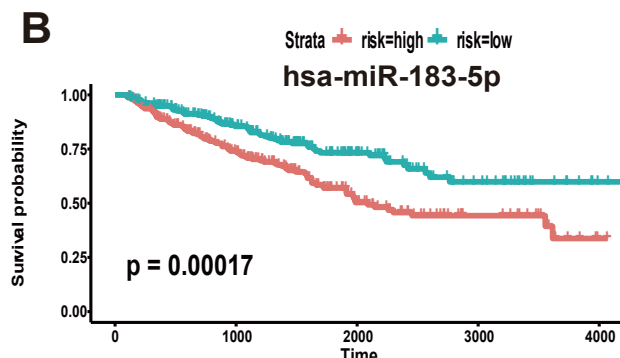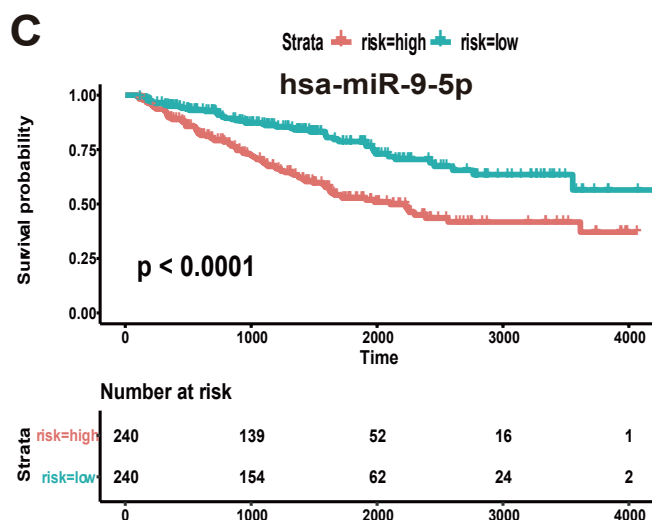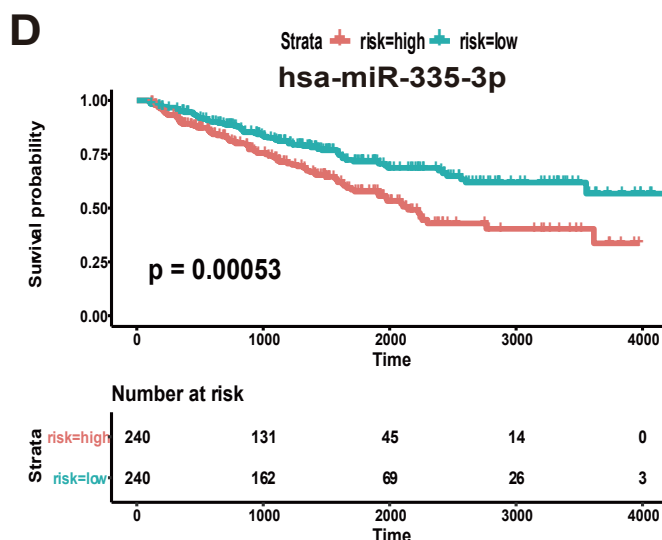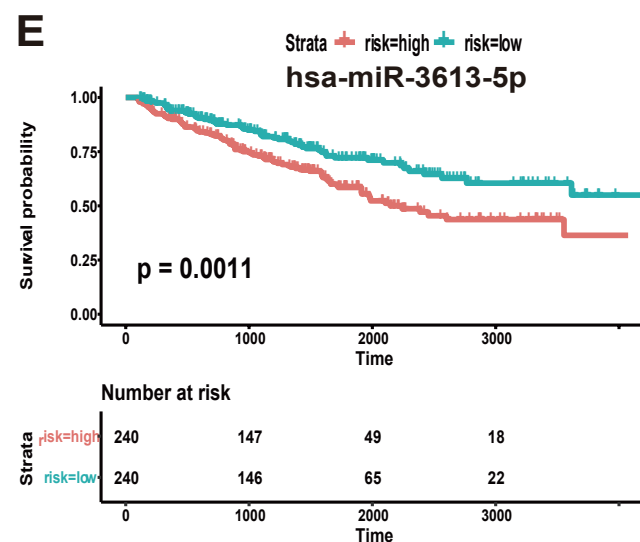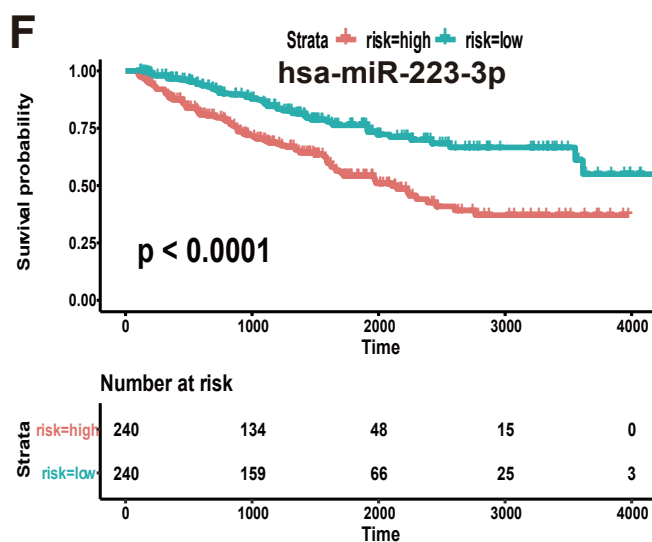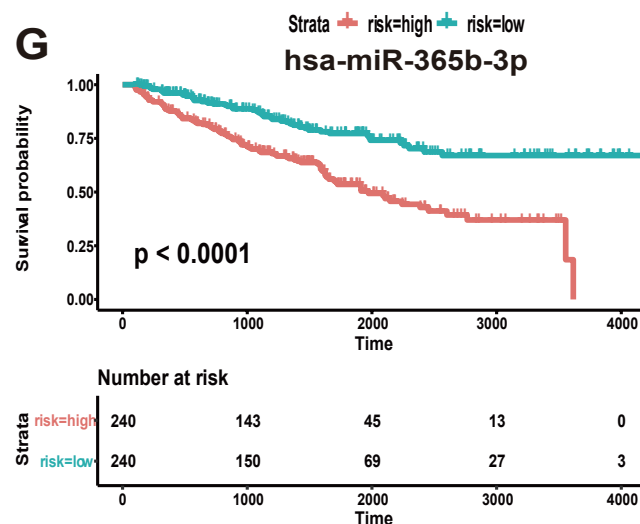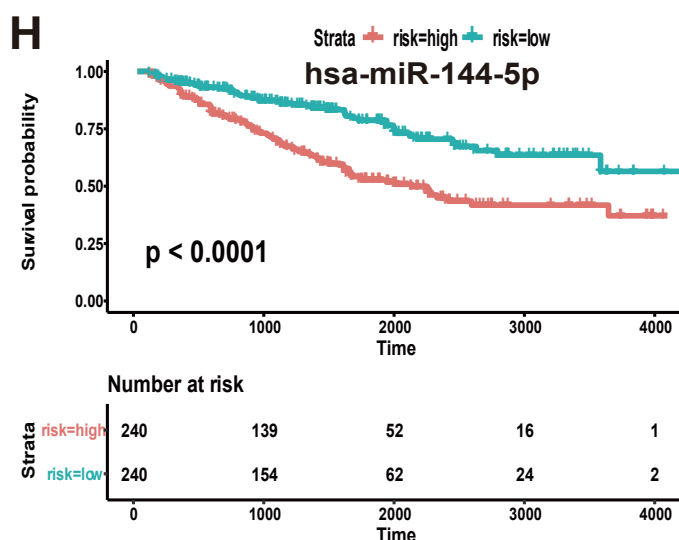

Supplement: Supplementary file 1 [file cancers-11-01668-s001.zip › Figure S2.pdf]

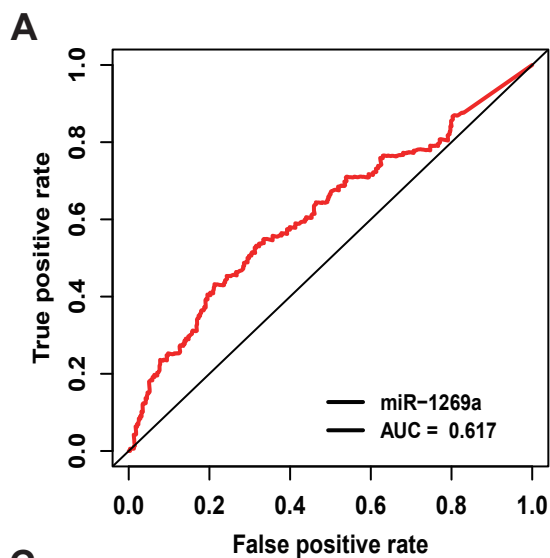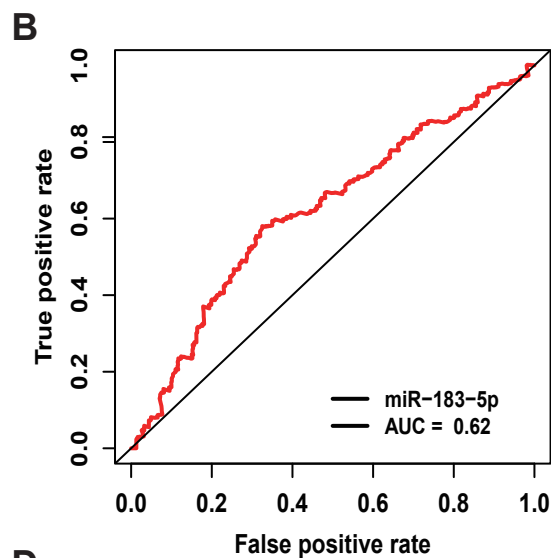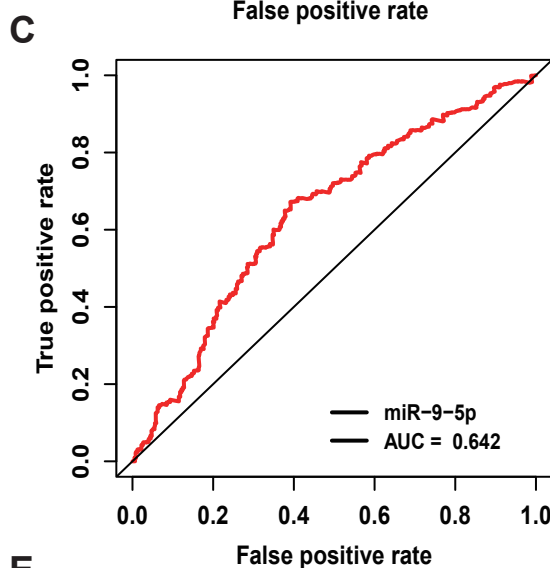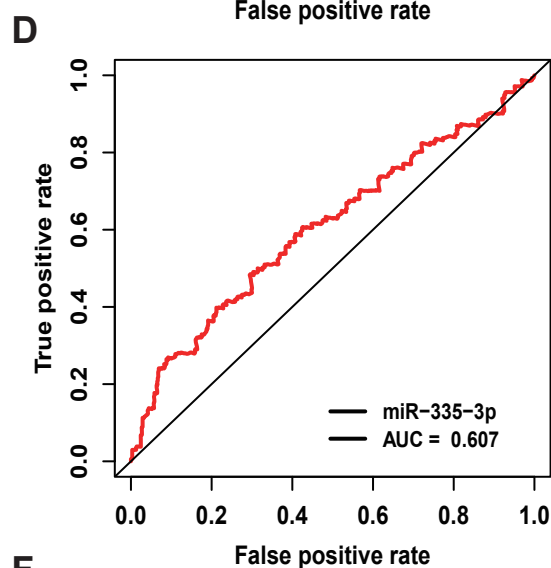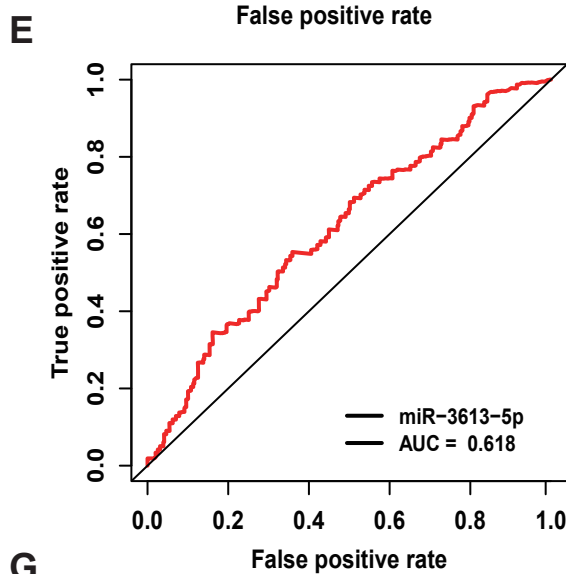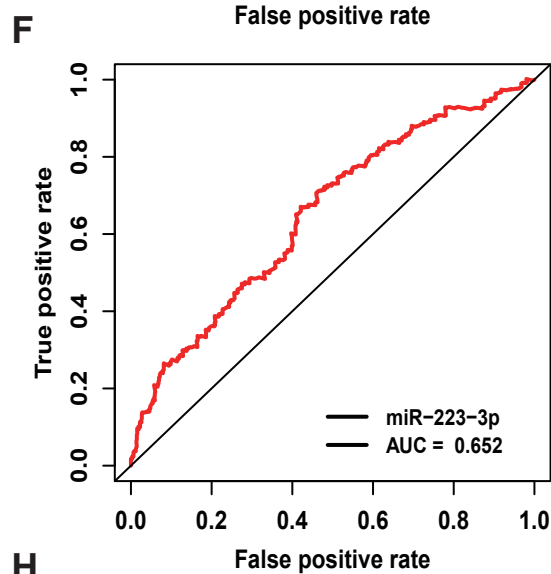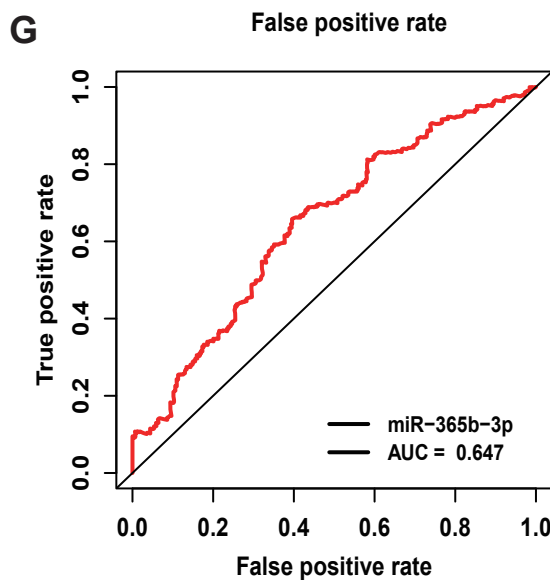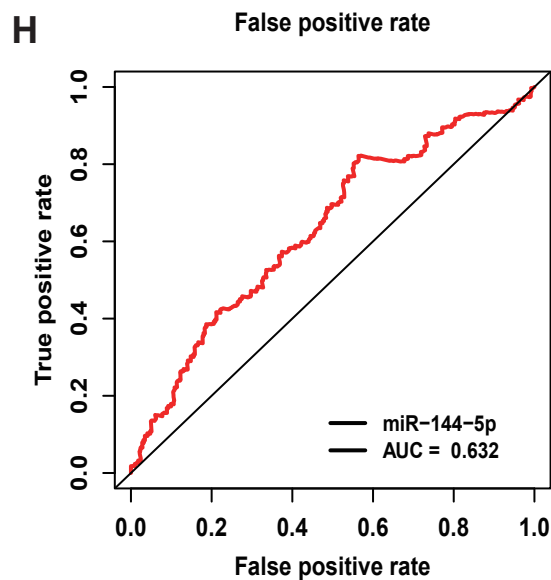

Supplement: Supplementary file 1 [file cancers-11-01668-s001.zip › Figure S3.pdf]

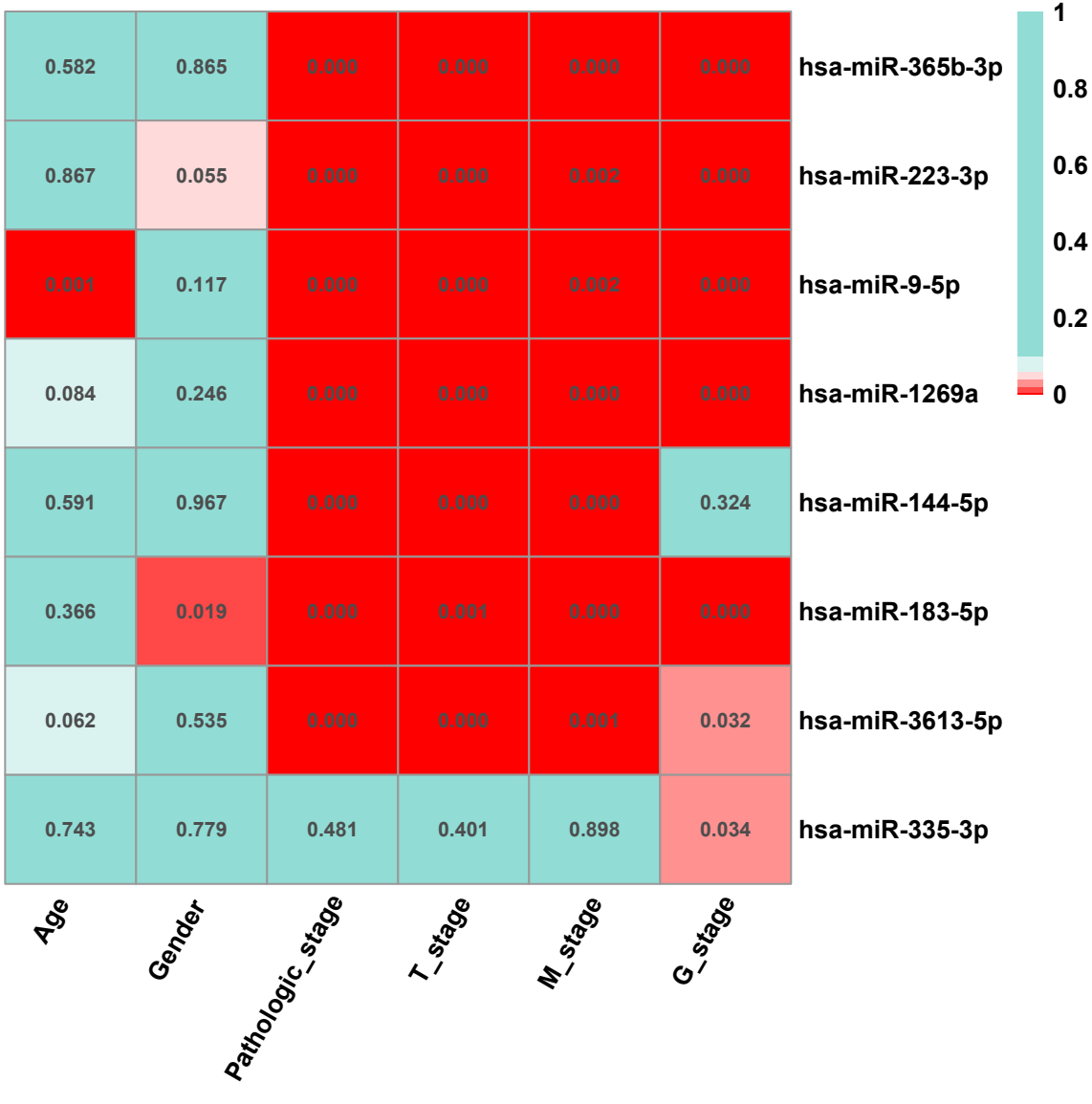

Supplement: Supplementary file 1 [file cancers-11-01668-s001.zip › Figure S4.pdf]

**A**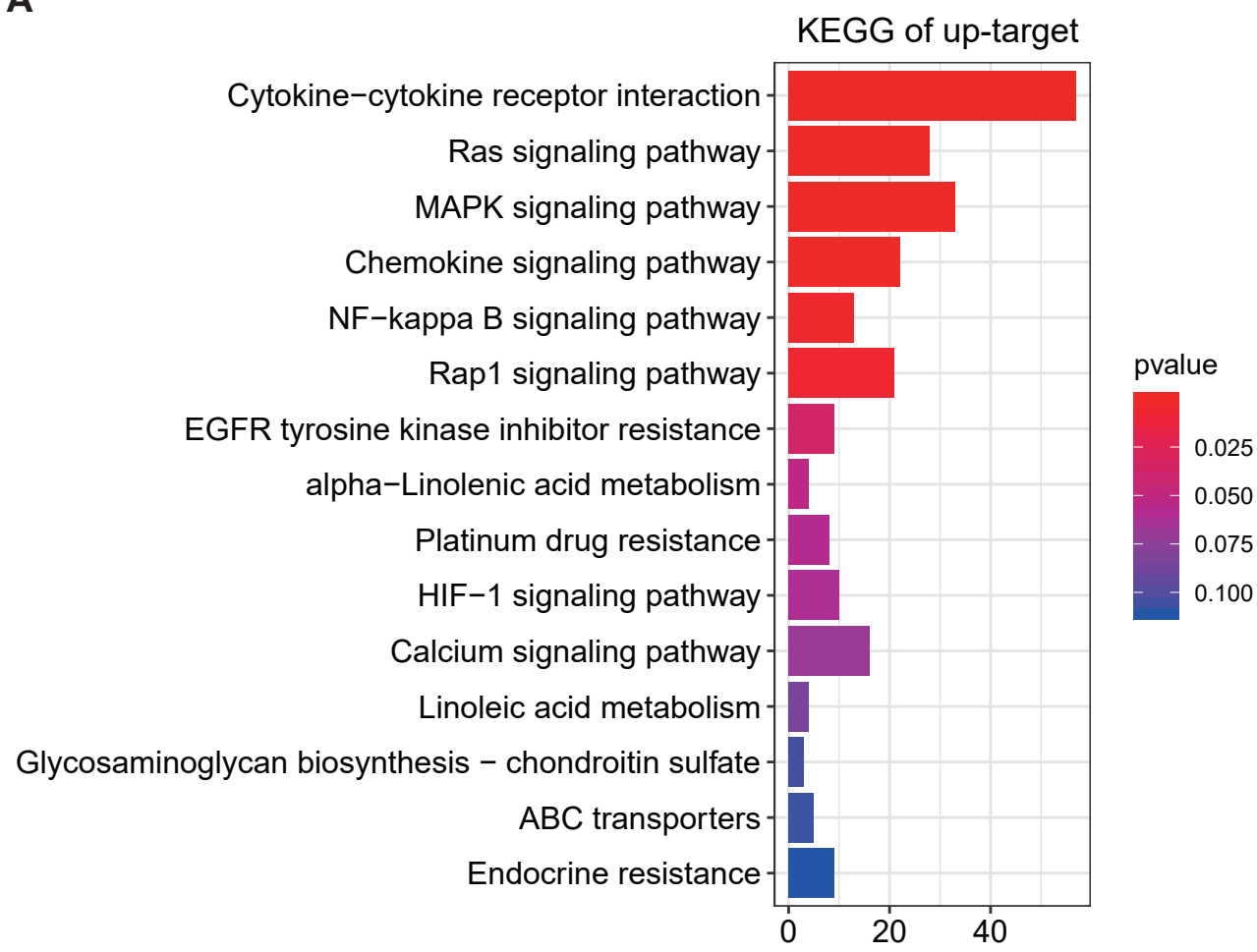**B**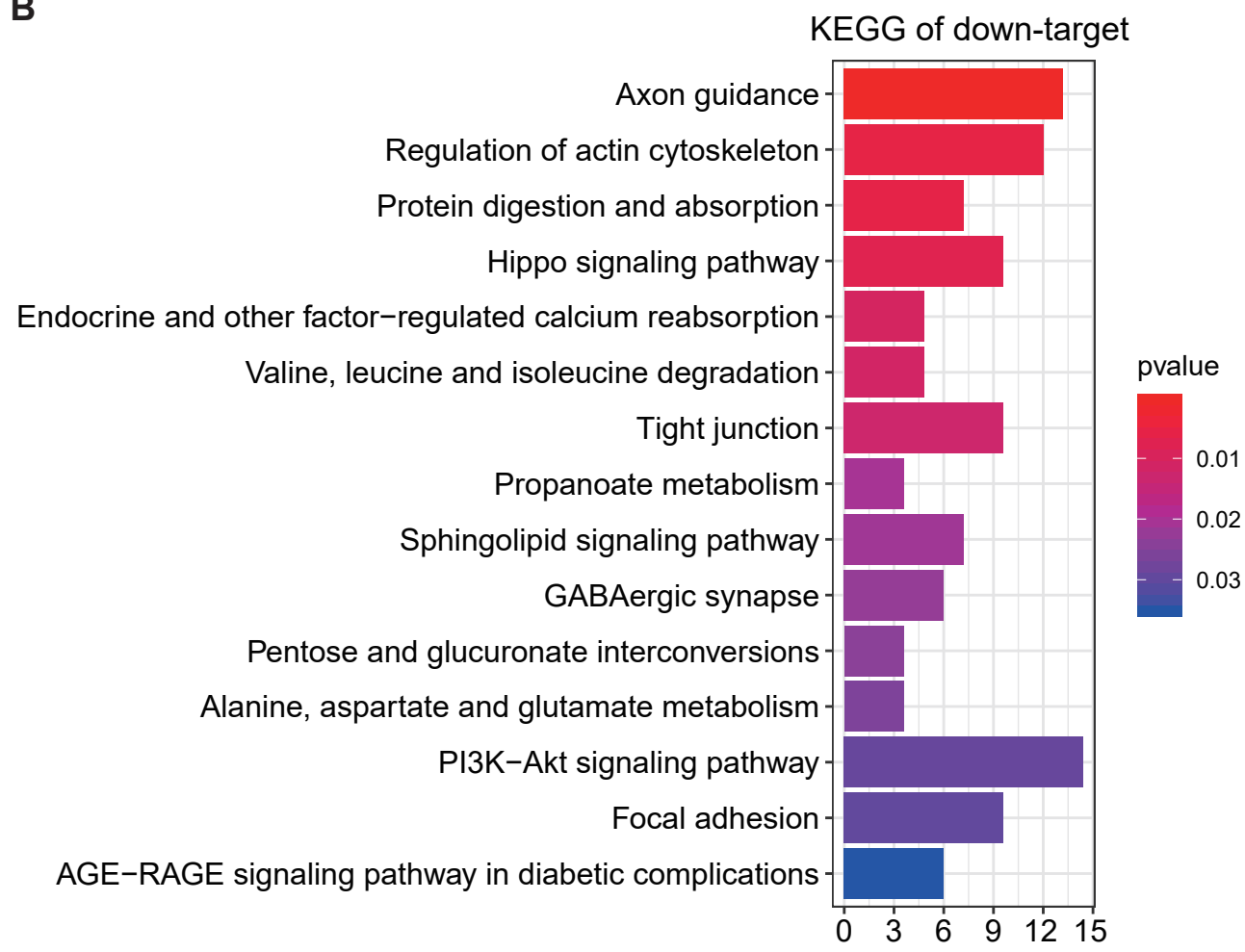

Supplement: Supplementary file 1 [file cancers-11-01668-s001.zip › Figure S5.pdf]

**A**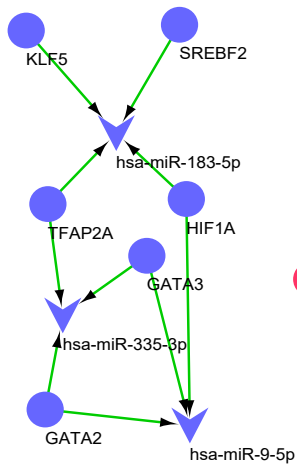**B**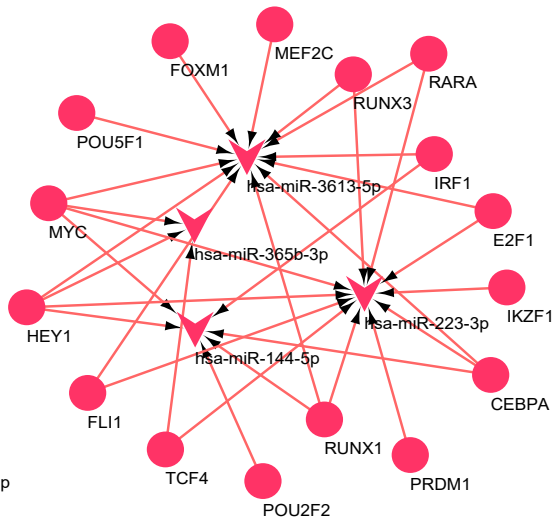

Supplement: Supplementary file 1 [file cancers-11-01668-s001.zip › Figure S6.pdf]
